# Socio-demographic, clinical and psychological predictors of healthcare utilization among patients with musculoskeletal disorders: a prospective cohort study

**DOI:** 10.1186/s12913-020-05100-0

**Published:** 2020-03-23

**Authors:** Cecilie Rud Budtz, Søren Mose, David Høyrup Christiansen

**Affiliations:** 1 Regional Hospital West Jutland, Department of Occupational Medicine, University Research Clinic, Gl. Landevej 61, 7400 Herning, Denmark; 2 VIA University College, School of Physiotherapy, Gl. Struervej 1, 7500 Holstebro, Denmark; 3 Department of Clinical Medicine, Health, Aarhus University, Palle Juul-Jensens Boulevard 82, 8200 Aarhus, Denmark

**Keywords:** Clinical course, Clinical pathway, Healthcare utilization, Physiotherapy, Prediction, Cohort study

## Abstract

**Background:**

Musculoskeletal disorders are common in the general population and a leading cause for care seeking. Despite the large number of patients with musculoskeletal disorders seeking care, little is known of the clinical course, pathways and predictors of healthcare utilization among these patients. The purposes of the study were to 1) describe the clinical course and related healthcare utilization in primary care physiotherapy and secondary healthcare among patients with neck, shoulder and low-back pain treated in physiotherapy practice, and 2) identify independent clinical, socio-demographic, psychological and general health predictors of healthcare utilization.

**Methods:**

The study was a prospective cohort study of patients seeking physiotherapy treatment for neck, shoulder, or low-back pain in physiotherapy practices across Denmark. A total of 759 physiotherapy patients completed questionnaires containing information on clinical course and potential predictors of healthcare utilization. Healthcare utilization was obtained from the Danish National Health Service Register and National Patient Register. Associations between potential predictors and low/high primary care physiotherapy utilization and hospital contacts in relation to specific neck, shoulder or low-back disorders were analysed using binomial regression analyses and adjusted for age, sex, duration of pain and comorbidity.

**Results:**

During 6 months follow-up, patients experienced clinically relevant improvements in pain, fear avoidance and psychological wellbeing. Patients with higher baseline pain and disability and who were on sickness leave were more likely to have high primary care physiotherapy utilization. Hospital contacts were predicted by higher levels of pain, disability and low psychological wellbeing.

**Conclusions:**

Clinical factors and sickness leave seems to be the main predictors of primary care physiotherapy utilization, whereas for secondary care contacts, psychological factors may also be of importance. The study contributes to the on-going research into clinical pathways and may identify future target areas to reduce healthcare utilization in patients with musculoskeletal disorders.

## Background

Musculoskeletal pain and disability are common in the general population and it has been estimated that over 1.2 billion people worldwide are affected by musculoskeletal disorders [1]. Musculoskeletal disorders are one of the leading causes for care seeking in primary care [2] and healthcare costs related to musculoskeletal disorders are enormous. It is estimated, that between 5.4 and 12.6% of all health expenditures in high-income countries are attributed to musculoskeletal disorders [3], and in Denmark low back pain is the single largest contributor to primary healthcare costs followed by neck pain as the second largest [4].

According to clinical practice guidelines the majority of the patients with non-specific musculoskeletal disorders should be managed and treated in primary care. In Denmark, most people with musculoskeletal disorders seeking care is referred to physiotherapy by their general practitioner (GP) and physiotherapy treatment is often a central part of the clinical pathway [5, 6]. The physiotherapy treatment should include physical examination, patient education, reassurance about a favourable prognosis, active management strategies and advice on returning to normal activities as well as exercise therapy [6]. Most of the patients treated in primary care physiotherapy will experience significant and clinically relevant improvements in outcomes such as pain intensity and disability within the first few weeks or months [7, 8]. However, we have limited knowledge on the level of primary care physiotherapy utilization associated with such improvements. In addition, it is largely unknown how many of these patients require referral for further evaluation in a hospital because of musculoskeletal conditions.

With limited healthcare resources an increasing interest on healthcare utilization and subsequently healthcare costs has naturally emerged. Thus, identifying predictors of healthcare utilization in both primary and secondary care could help allocate limited healthcare resources towards patients who are most in need. In the general population musculoskeletal pain and disability, as well as psychological factors, have shown to be predictive of both primary and secondary healthcare utilization [9–15]. Nevertheless, predictors of healthcare utilization among musculoskeletal physiotherapy patients are less well studied as research in this field mainly have focused on clinical outcomes such as pain or disability [16, 17]. A newly published study however concluded that baseline and 4-week changes in pain intensity, disability and pain-related psychological distress predicted self reported use of painkillers, injections, surgery, diagnostic testing and emergency room visits among physiotherapy patients [11]. The challenge with self reported survey data is that the results can be biased due to recall or loss to follow up [18]. In Denmark, it is possible to use individual-based National Healthcare registries to identify healthcare utilization, thereby minimizing the risk of bias. National registry-data have never been used to investigate clinical, socio-demographic, psychological or general health factors as independent predictors of healthcare utilization in a population of physiotherapy patients. Thus, the objectives of the study were to 1) describe the clinical course and related healthcare utilization in primary care physiotherapy and secondary healthcare among patients with neck, shoulder and low-back pain treated in physiotherapy practice, and 2) identify independent clinical, socio-demographic, psychological and general health predictors of healthcare utilization.

## Methods

### Study design and population

The study was part of a large prospective cohort study evaluating the utility of standardized electronic data collection tools for patients referred to physiotherapy treatment for neck, shoulder, or low-back pain in 23 physiotherapy practices across Denmark from January to June 2016. All physiotherapy practices in Denmark (approximately 500 practices) were invited to participate in the project through an online invitation (distributed on the webpage www.praksis.dk) and a total of 26 practises applied for participation, of which three practises declined after further information about the project [19]. Patients were referred to physiotherapy treatment from their GP, and invited to participate if they were aged 18 years or above and able to understand Danish well enough to complete online questionnaires. No specific diagnostic criteria was applied other than neck, shoulder, or low-back pain presented at referral. No attempts were made to control treatment, which were left up to the treating physiotherapist discretion. The study was approved by the Danish Data Protection Agency (No. 2012–58-006). Under Danish law, this study did not need ethics approval (Act on Research Ethics Review of Health Research Projects, October 2013) [20].

### Data collection

All questionnaire and clinical data were collected using an existing web-based clinical database (www.fysdb.dk). Patients who agreed to participate in the study were asked to complete a questionnaire 1–2 days prior to their first physiotherapy consultation and at 3 and 6 months follow up. Participants were notified by e-mail when follow-up questionnaires were available for completion. Questionaries’ included items on occupational status, duration of pain, pain intensity, disability, pain behaviour, and psychological well-being which were measured using validated scales; the Numeric Pain Rating Scale (NRS) [21–23], the Neck Disability Index (NDI) [24], the Disabilities of the Arm, Shoulder and Hand (Quick DASH) [25], the Roland Morris Questionnaire (RMQ) [26], the Örebro Musculoskeletal Pain Screening Questionnaire [27, 28], and the WHO 5 Well-being Index [29]. At follow up patients also answered a single question in relation to the Patient Acceptable Symptom State (PASS) – i.e. the highest level of symptom at which patients find their condition acceptable [30]. The wording of the question was “*If you were to remain for the next few months as you are now, would you consider your current state to be satisfactory?*” The question was answered with yes or no. Furthermore, we included information on time of referral, pain site, comorbidity and private health insurance collected at first physiotherapist consultation.

### Outcome

Primary outcome was contacts in relation to primary and secondary healthcare utilization services obtained from the Danish National Health Service Register (NHSR) [31] and National Patient Register (NPR) [32]. The NHSR contains week-by-week information on physiotherapy interventions received in private primary care since 1990 with the exception of self-paid therapy without reimbursement. The NPR includes information on diagnosis, hospital and contact dates for all in- and outpatient contacts in public and private somatic hospitals in Denmark. In NPR, diagnoses are coded using the International Classification of Diseases and Related Health Problems (ICD-10) system [33].

#### Primary care physiotherapy

We extracted all NHSR records of physiotherapy contacts for each patient within a 6 months period from their baseline questionnaire date. As physiotherapy contacts did not follow a normal distribution and to ease interpretation of the results, the total number of contacts (first consultation, individual treatment session or group exercise) was dichotomized into few (< 6 contacts) or many (≥ 6 contacts). The individual treatment sessions would most often include a combination of exercise therapy, manual therapy, instruction/advice on home exercise and to a limited extend physical modalities [8, 34]. The chosen cut off level of 6 contacts were based on the median number of treatments in a previously conducted study in Danish primary care physiotherapy [8].

#### Secondary care

From NPR we extracted all records of hospital contacts for each patient within a 12 months period from their baseline questionnaire date. Relevant diagnose codes in chapter XIII: *Diseases of the musculoskeletal system and connective tissue* and the first part of chapter XIX: *Injury, poisoning and certain other consequences of external causes* were included. (For details on diagnose codes see Appendix 1). Secondary care contacts were dichotomized into contact (≥ 1 specific diagnose) or no contact during 12 months follow-up. As it can be difficult to clinically distinguish between low back and neck disorders, patients referred for physiotherapy with low back or neck pain were classified as having a related secondary healthcare contact if they had an ICD-10 diagnose code related to either low back or neck disorders. Similar, shoulder patients were classified as having a contact if they had an ICD-10 diagnose code related to either shoulder or neck disorders as patients referred with neck disorders clinically could present as a patient with shoulder pain.

### Potential predictors of healthcare utilization

Based on previously conducted studies [10, 11], potential predictors of healthcare utilization from the following four health domains were included:

- *Clinical factors: Pain intensity* was assessed as average pain the preceding week on a NPRS scale ranging from 0 “No pain” to 10 “worst pain imaginable” [21–23], and *disability* which included region-specific disability questionnaires (RMQ [26], NDI [24] and Quick-DASH [25]). As the scale structures of these questionnaires are very different the scores were standardised by nearest centile and converted into a 0–100 score, with 100 being the highest level of disability to allow scores to be fitted into the same regression model. We also included *pain site* (low back, neck or shoulder) representing the reason for referral.
- *Socio-demographic factors: Level of education* (years of education after compulsory schooling. Education was categorized into three: None, lower (< 3 years) or vocational and training, or medium level (3–4 years) / higher level (> 4 years), *sickness leave* (patient was asked by the physiotherapist at first consultation), and *private health insurance* (patient was asked by the physiotherapist at first consultation).
- *Pain behaviour and psychological factors:* Included *fear avoidance* assessed by two questions from the Danish version of the Örebro Musculoskeletal Pain Screening Questionnaire (ÖMPSQ) [27, 28, 35–37]. Each question was scored on a 0–10 scale, and added to a sum score from 0 (no fear avoidance) to 20 (high fear avoidance), and *psychological wellbeing* on a scale from 0 (low wellbeing) to 100 using the WHO Wellbeing Index (WHO-5) which is a five-item questionnaire assessing subjective psychological wellbeing [29].
- *General Health* consisted of a single question of the SF-36 questionnaire: “In general, would you say your health is” with five response categories on a Likert scale addressing the patients’ perception of their general health status [38]. Because of few responses in the poor and excellent category (*n* < 5), the five-point scale was reduced to three categories 1) excellent/very good, 2) good and 3) fair/poor.

### Other existing prognostic factors

To investigate if the potential predictors contributed independently to the prediction of healthcare utilization, analyses were adjusted for the following known non-modifiable prognostic factors [11]: *1) Age*, 2) *gender*, 3) *duration of pain* (based on baseline questionnaire), which was dichotomized into under/over 3 months and 4) *comorbidity* (patients were asked at the first consultation if they had other health problems), which was dichotomized into comorbidity or no comorbidity.

### Statistical analysis

Differences between participants versus non-participants and responders versus non-responders to follow up questionnaires were analysed using two-sample t test for continuous variables and Pearson chi squared for dichotomous variables. Descriptive statistics (percentages, means) were used to report missing values, the clinical course and healthcare utilization. Changes in pain, fear avoidance and psychological wellbeing for patients with few or many primary care physiotherapy contacts were presented graphically and differences between analysed using two-sample t-test with equal variances. Contact/no contact to secondary care in relation to specific shoulder, neck or low back pain disorders were analysed using two-sample t test with equal variances.

Associations between each potential predictive variable and few/many contacts to primary care physiotherapy or contact to secondary care were analysed using binomial regression analyses and adjusted for age, gender, duration of pain and comorbidity. Prior to the analyses log odds linearity assumptions for binomial regression analyses were controlled. Categorized variables were collapsed if there were fewer than 10 patients in a category. No formal sample size calculation was performed, but considerations on the number of potential variables to include in multivariable analyses were based on the principle of at least 10 cases per variable [39, 40].

Robustness of results were assessed by sensitivity analyses using 10 contacts to primary care physiotherapy as an alternative cut-off point and any contact to secondary care related to ICD-10 chapter XIII as alternative outcomes.

All statistical analyses were performed using STATA version 15.0 (StataCorp LP, College Station, TX, USA).

## Results

### Baseline characteristics

**Fig. 1:**
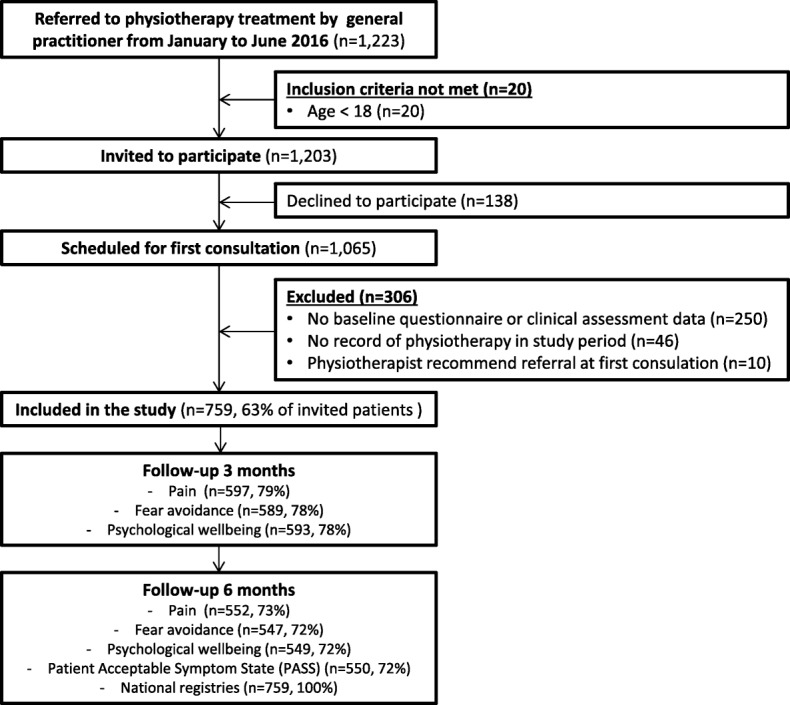
Flow of the participants through the study

The flow of participants is presented in Fig. 1. A total of 1203 patients met the inclusion criteria and after exclusion, 759 patients were included (63% of invited patients).

Baseline characteristics of the patients are presented in Table 1. Excluded patients (*n* = 306) were younger than the patients included in the study with mean difference being 2.3 years (95% CI 0.4; 4.3), whereas gender was equally distributed between included and excluded patients. Missing values ranged from 1% (occupational status and level of education) to 11% (comorbidity), with the majority of variables having less than 2% missing values.

**Table 1:** Baseline characteristics (*n* = 759)

Variable	
Sex, n (%)	
Female	436 (57)
Male	323 (43)
Age, mean (SD)	50 (14.5)
Occupational status, n (%)	
Employed	489 (65)
Unemployed	31 (4)
Retired/early retirement/flex job/disability pension	186 (24)
Student/on leave	44 (7)
Sickness leave, n (%)	82 (11)
Comorbidity, n (%)	290 (43)
Pain duration, n (%)	
> 3 months	418 (55)
< 3 months	341 (45)
Pain site, n (%)	
Low back	329 (43)
Neck	206 (27)
Shoulder	224 (30)
Pain 0–10, mean (SD)	6.2 (2.1)
Standardized disability 0–100, mean (SD)	52.2 (27.6)
Fear avoidance 0–20, mean (SD)	10.9 (5.3)
Psychological wellbeing (WHO-5) 0–100, mean (SD)	56.8 (20.4)

*Abbreviations*: *SD* Standard deviation, *WHO* World Health Organization

### Clinical course and healthcare utilization

Patients who were lost to follow up (non-responders) during 6 months were more often women, were younger (mean difference 3 years (95% CI 0.7; 5.3)), had higher baseline pain scores (mean difference 0.5 (95% CI 0.1;0.8)) and worse psychological wellbeing (mean difference 5.8 (95% CI 2.6;9.1)). Among patients with follow up (responders) the average decrease in pain was − 2.8 points (95% CI -3.1; − 2.6) corresponding to a 45% improvement from baseline to 6 months follow up. Slightly lower improvements were observed for fear avoidance and psychological wellbeing with an average improvement of − 2.2 points (95% CI -2.7; − 1.7) (20%) and 12.7 points (95% CI 11.0; 14.4) (22%) respectively. At 6 months, 56% of the patients perceived their symptoms as satisfactory on the PASS. For a period of 6 months from baseline, the median number of contacts in primary care physiotherapy was 5 [Interquartile Range (IQR) 3 to 9]. A total of 577 patients (72%) had an individual first consultation and 553 of these patients also had one or more individual treatment sessions (e.g. exercise therapy, manual therapy or instruction/advice). A total of 65 patients (8.6%) also received group exercise with the highest prevalence among patients with low back pain (11%) and the lowest prevalence among patients with neck pain (5%). The median number of group exercise was 6 [IQR 4 to 10]. For a period of 12 months from baseline, 112 patients (15%) had a contact related to ICD-10 chapter XIII: Diseases of the musculoskeletal system and connective tissue. A total of 84 patients (11%) had one or more hospital contacts related to specific low back, neck or shoulder disorders during the follow-up period. No difference in healthcare utilization was detected between responders and non-responders (data not shown).

**Fig. 2:**
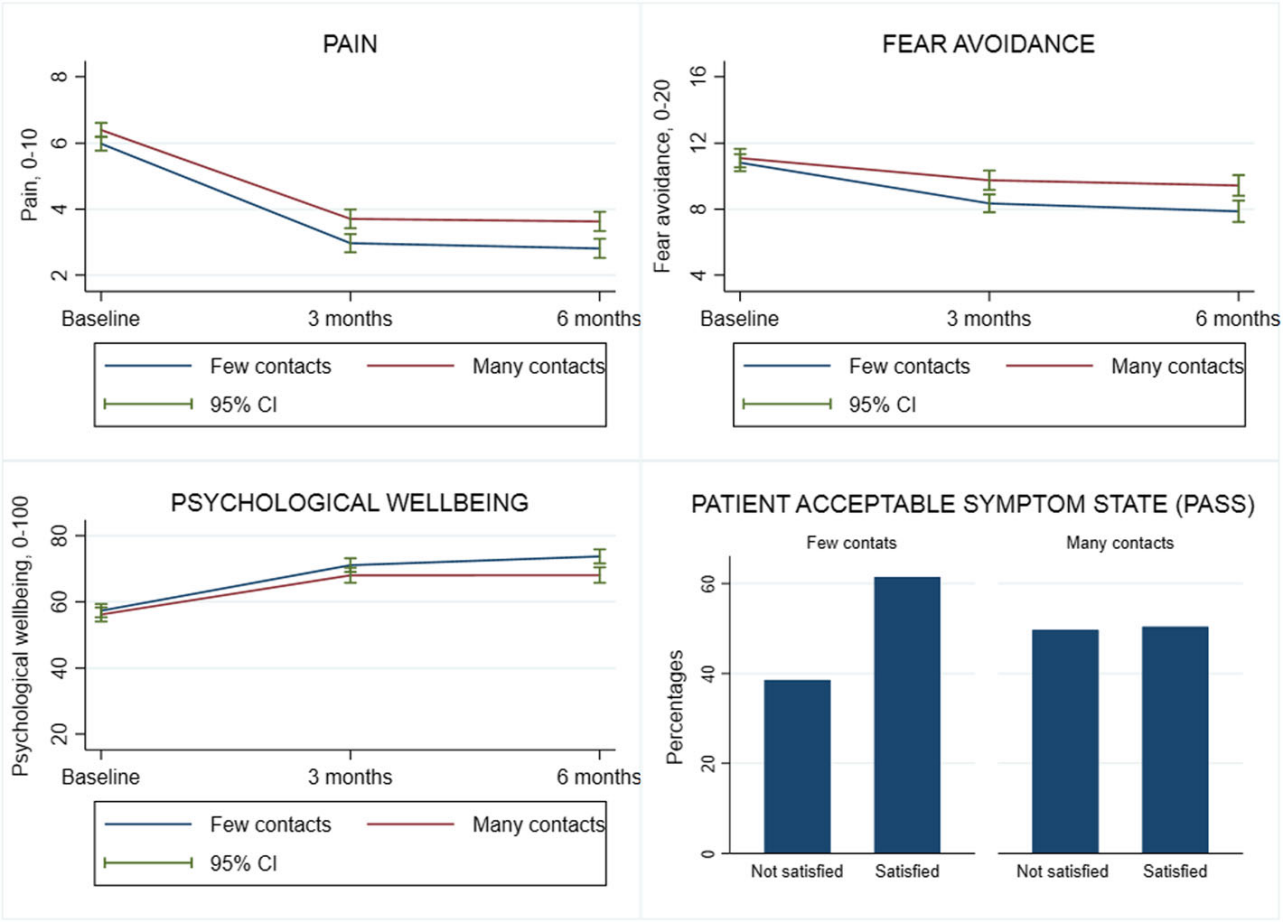
Changes in pain, fear avoidance and psychological wellbeing from baseline to 6 months follow-up and Patient Acceptable Symptom State at 6 months in patients with low back, neck or shoulder pain

Figure 2 depicts changes from baseline to 6 months follow up in pain, fear avoidance and psychological wellbeing and PASS at 6 months with respect to few or many primary care physiotherapy contacts. On average, patients with few contacts had significantly greater improvements in fear avoidance (mean difference − 1.15 (95% CI -2.18; − 0.12) and psychological wellbeing (mean difference 4.91 (95% CI 1.55; 8.26) than patients with many contacts. Taking baseline levels into account, patients with few contacts experienced a 26% improvement in both fear avoidance and psychological wellbeing compared to patients with many contacts who experienced improvements of 14% in fear avoidance and 18% in psychological wellbeing.

### Prediction of healthcare utilization

Three predictors of high primary care physiotherapy utilization were identified (Table 2). Patients with higher pain, disability and who were on sickness leave were more likely to have six or more primary care physiotherapy contacts. Changing the cut-off point from 6 to 10 primary contacts had little impact on the identified associations. Patients with higher pain, disability and psychological wellbeing scores were more likely to have a contact to secondary care. Changing secondary care outcome to merely being a contact related to ICD-10 chapter XIII: *Diseases of the musculoskeletal system and connective tissue* did not influence the already identified associations, but a further two associations were identified; fear avoidance and general health. See Appendix 2.

**Table 2:** Associations (OR) between potential predictors and healthcare utilization (*N* = 759)

Potential predictor	Contact with primary care physiotherapy in relation to low-back, shoulder or neck pain					Contact with secondary care in relation to specific low-back, neck or shoulder disorders				
	Contacts		Crude	Adjusted^**d**^		Contacts		Crude	Adjusted^**d**^	
	Cases^b^ (n)	%	OR	OR	95% CI	Cases^c^ (n)	%	OR	OR	95% CI
**Clinical**										
Pain 0-10^a^	352	46	1.10	1.09	(1.01;1.17)*	84	11	1.24	1.26	(1.11;1.44)*
Standardized disability 0-100^a^	347	46	1.01	1.01	(1.00;1.02)*	82	11	1.02	1.02	(1.00;1.03)*
Pain site										
Low back	141	43	1.00	1.00	–	37	11	1.00	1.00	–
Neck	106	51	1.41	1.18	(0.81;1.74)	17	8	0.71	0.73	(0.38;1.41)
Shoulder	105	47	1.18	1.03	(0.71;1.50)	30	13	1.22	1.13	(0.64;1.99)
**Socio-demographic**										
Level of education										
None	56	43	1.00	1.00	–	15	12	1.00	1.00	–
Low (< 3 years) or vocational and training	182	47	1.17	1.05	(0.67;1.63)	48	12	1.09	1.31	(0.64;2.67)
Middle (3–4 years) or high (> 4 years)	111	47	1.17	0.99	(0.62;1.59)	20	8	0.71	0.79	(0.36;1.76)
Sickness leave^e^										
No	296	45	1.00	1.00	–	69	10	1.00	1.00	–
Yes	46	56	1.57	1.74	(1.03;2.94)*	14	17	1.76	1.87	(0.91;3.82)
Private Health Insurance										
No	215	44	1.00	1.00	–	58	12	1.00	1.00	–
Yes	115	50	1.30	1.32	(0.93;1.87)	20	9	0.72	0.73	(0.41;1.33)
**Psychological**										
Fear avoidance 0-20^a^	345	46	1.01	1.02	(0.99;1.05)	83	11	1.05	1.03	(0.98;1.08)
Psychological wellbeing (WHO-5) 0-100^a^	350	47	1.00	1.00	(0.99;1.00)	84	11	0.99	0.99	(0.97;1.00)*
**General**										
General Health										
Excellent/very good	128	42	1.00	1.00	–	31	10	1.00	1.00	–
Good	156	49	1.36	1.27	(0,90;1.79)	33	10	1.04	0.94	(0.53;1.67)
Fair/poor	67	51	1.47	1.14	(0,71;1.82)	20	15	1.61	1.82	(0.92;3.61)

*Abbreviations*: *OR* Odds ratio, *CI* Confidence interval, *WHO* World Health Organization

* *p* < 0.05

^a^OR per 1 unit increase in score

^b^patients with ≥6 contacts to primary care physiotherapist

^c^patients with ≥1 contact to secondary care

^d^Adjusted for age, gender, duration of pain (under/over 3 months) and comorbidity (yes/no)

^e^Patients retired or with flex job or on disability pension was also included in this analysis (*n* = 186)

## Discussion

This study evaluated the clinical course, healthcare utilization and predictors of healthcare utilization among patients with neck, shoulder or low back pain treated in Danish physiotherapy practice. During 6 months follow-up, patients experienced clinically relevant improvements in pain, fear avoidance and psychological wellbeing. However, only 56% of the patients rated their symptoms as acceptable at 6 months. On average, patients with few contacts had significantly greater improvements in fear avoidance and psychological wellbeing than patients with many contacts. During 6 months from baseline the median number of treatments in primary care physiotherapy was 5 [IQR 3 to 9] and during 12 months 11% of the patients had a secondary healthcare contact related to specific neck, shoulder or low back disorders. Three predictors were identified for primary care physiotherapy utilization: Higher levels of pain and disability and sickness leave and three predictors emerged for secondary care contacts: Higher levels of pain and disability and psychological wellbeing.

### Limitations and interpretation

A limitation in the study was the modest follow-up rates at 3 and 6 months in questionnaire data and as differences between responders and non-responders were identified we cannot exclude differential attrition bias with respect to the results of the clinical course. However, the estimated differences in pain and psychological wellbeing were small and no subsequent difference in healthcare utilization was detected between responders and non-responders, thus the risk of bias of the estimated clinical course is considered limited. As healthcare utilization was based on national health registries with 100% follow-up attrition bias in these analyses are not present. Still, when using registry data there is a risk of bias due to misclassification. Such misclassification would not be associated with any specific exposure group and therefore most likely to be non-differential thereby risking bias towards no association [41]. Clinical course and potential predictors of healthcare utilization relied on patient self-reported questionnaire data. Although we used validated questionnaires, misclassification and missing values may have affected our results. Such misclassifications would also be non-differential and missing values were few and could only have minor effect on the result. The potential predictor *fear avoidance* was measured using two questions from ÖMPSQ [27, 35, 37]. The subscale of fear avoidance has previously shown to be predictive of poor outcomes in patients with musculoskeletal disorders [8, 42–44]. Although these earlier findings have used a subscale of three fear avoidance questions from the original 21 item version of ÖMPSQ, we chose only to use the two questions from the shortened 10 item version of ÖMPSQ [35, 36], since it has shown to be as predictive as the longer version [35].

The results were naturally influenced by cut-off values in healthcare utilization. Nevertheless, changing cut-off values of low/high primary care physiotherapy had little impact on the estimated associations. Grouping according to more specific ICD-10 diagnose codes of neck, shoulder or low back conditions resulted in a very small group of patients (cases). This lead to limited statistical power and although the estimated association on e.g. general health was strong (adjusted OR 1.82 (95%CI 0.92; 3.21), the association did not reach statistical significance. Moreover, when the outcome was changed to contacts of overall diseases of the musculoskeletal system and connective tissue, thereby increasing the number of cases, associations with general health was statically significant (OR 1.86 (95%CI 1.00;3.45). This underpins the importance of general health perceptions, when predicting secondary healthcare utilization. Larger studies could advantageously explore this association further.

#### Clinical course and healthcare utilization

The observed improvements in pain were similar to those reported among Danish musculoskeletal physiotherapy patients [8] and low back pain patients [7]. Although, such improvements are considered clinically worthwhile by patients [45], these results cannot be interpreted as a study on the effectiveness of physiotherapy as considerable improvements in pain has been observed without any treatment [46]. The more modest improvements in fear avoidance and psychological wellbeing may imply that treatment of psychological factors may be inherently difficult in primary care physiotherapy. This is to some extend supported by the significantly lower improvements in fear avoidance and psychological wellbeing observed for patients with high levels primary care physiotherapy utilization in our study. On the other hand although these differences reached statistical significance, between group differences were small and the clinical relevance of these findings may be questioned. Another result that suggests effective treatment of musculoskeletal pain is a very challenging and difficult task is the fact that only 56% of the patients rated their symptoms as acceptable at 6 months follow-up. This result resembles findings from a similar previous conducted study, where 52% of the patients rated their symptoms as acceptable at 6 months follow up in a similar population [8]. The reason for this modest level of acceptability among patient with musculoskeletal disorders is unknown, but it would be very interesting to further explore whether the unsatisfied patients perhaps seek treatment elsewhere or went back to their GP. The median number of treatments in primary care physiotherapy was 5 [IQR 3 to 9] in our study. This result is similar to existing results on number of contacts during an episode of physiotherapy care, where the previously reported median number of was 6 [IQR 3;10] [8]. Other studies have shown means of 5.5 (SD 2.5) visits [47] and 7.1 (SD 12.2) visits [48], hence the median in the present study seems to in line with the existing evidence. In Denmark, patients are referred by their general practitioner to physiotherapy treatment, but as it is the case in many countries, an increased interest on and advocate for direct access to physiotherapy are emerging. This is among other things advocated as a possible way to ensure a better clinical outcome and reduce the number of contacts to the physiotherapists [49, 50]. When looking at the distribution of the contacts only 65 (8.6%) patients received group exercise, hence the majority of treatment was individual based. This may be the result of physiotherapist preference, clinical reasoning or patients’ not requesting group exercise. Nevertheless, the improvement in pain intensity, psychological wellbeing and fear avoidance of these patients was reached as a result of a reasonably limited number of treatments in primary care physiotherapy. For a period of 12 months from baseline, 84 patients (11%) had one or more hospital contacts related to specific low back, neck or shoulder disorders. Previously conducted studies examining healthcare utilization among physiotherapy patients have been based on health insurance databases [47] or the Military Health System [48] in the United States, thereby risking biased results due to selection. A direct comparison between the studies is challenging as healthcare utilization is defined differently between the studies and vary between diagnostic, surgical or injection procedures. In our study, we have only focussed on diagnose in a secondary care setting. It would be highly valuable to further explore which procedures and actions the patients encountered in secondary care, as there is an increased interest on unwarranted procedures among patients with musculoskeletal disorders [51, 52]. To our knowledge, no other studies have examined this direct link between primary care physiotherapy and secondary care contacts in relation to specific diagnoses using registry data. The relatively small proportion of patients that are ultimately referred for secondary care because of specific neck, shoulder or low back disorders indicates that patients with musculoskeletal pain to a large extend are managed and treated in primary care as recommended [6].

#### Predictors of healthcare utilization

Three independent predictors of high primary care physiotherapy utilization were identified: higher levels of pain and disability and sickness leave. These results add to the on-going research into predictive factors of healthcare utilization and to our knowledge no other studies have investigated independent clinical, socio-demographic, psychological and general health predictors of high primary care physiotherapy utilization among physiotherapy patients. However, these results are in line with large population-based cohorts examining predictive factors of healthcare utilization [9, 10, 12], and it seems that the same predictive factors may be important among the general population as well as a population of patients with musculoskeletal disorders. Interestingly high utilization of primary care physiotherapy seems to be predicted by these classical clinical factors and sickness leave and not psychological or general health factors, which were not associated with more physiotherapy contacts. This could imply that physiotherapists are generally still treating musculoskeletal pain from a biomedical perspective, as physiotherapists seem to increase the number of treatment according to clinical factors and not psychological factors. These results could suggest that the advocated bio-psycho-social approach to these patients [52, 53] is not yet well implemented in physiotherapy practice. On the other hand, patients with few physiotherapy contacts also showed greater improvements in psychological wellbeing and fear avoidance compared to patients with many contacts. This could imply that the physiotherapist indeed addresses these factors and patients who are able to adhere to the physiotherapists’ advice experience greater improvements. Adding to that implication, it should also be noted that psychological factors seems to be important when predicting secondary care utilization, thereby suggesting that patients who need a more comprehensive intervention are ultimately referred for secondary care. These possible explanations for the found predictors could be very interesting to explore further in future research. Other predictors of secondary care contact were also higher levels of pain and disability and these results are in line with identified predictors of secondary care contacts in a similar population [11]. The results suggests, that there is a need for treatment options to address psychological factors and pain behaviour among patients with musculoskeletal disorders not only in primary care but also in secondary care. The results on independent predictors of healthcare utilization are an important first step into future prognostic research in this area [54, 55]. The present study indicates which factors is of importance when predicting healthcare utilization and thus future studies could build on these results to develop stratified clinical pathways. The focus on stratified treatment options is not new [56], and previously tools such as the Örebro Musculoskeletal Pain Screening Questionnaire [28] or the STarT Back Screening Tool [57] have been developed to identify patients at risk of chronicity, thereby ensuring the right treatment for the right patient at the right time. Our results forms a base for further prognostic model research, which could eventually help tailor treatment decisions, thereby shaping future clinical pathways ensuring that limited healthcare resources are allocated to those most in need.

#### Generalisability

As patients were recruited consecutively in 23 physiotherapy practices across Denmark, we believe the cohort to be representative of patients with neck, shoulder or low back disorders seen in primary physiotherapy practice. A limitation of the study was the participation rate at baseline (63%) which could affect the generalizability of our findings. Such non-participation rates are however common in large population studies and evidence from other Danish studies suggest the estimated associations may not necessarily be biased by non-participation [58, 59].

## Conclusion

When predicting future health care utilization among patients with musculoskeletal disorders it seems that the clinical factors and sickness leave is of importance to the utilization of primary care physiotherapy. This contrasts to secondary care utilization which seems to be predicted not only by clinical but also by psychological factors. The study contributes to the on-going research into clinical pathways and may identify future target areas to reduce healthcare utilization in patients with musculoskeletal disorders.

## Data Availability

The study dataset from which we have reported findings in this paper cannot be accessed by other researchers according to Danish regulations.

## Appendix 1

The following ICD-10 diagnose codes represented contacts related to low back, neck or shoulder disorders:

- *Low back*; M40-M54 (dorsopathies), M80–M85 (disorders of the bone density and structure), M99 (biomechanical lesions, not elsewhere classified), S32 (fracture of lumbar spine and pelvis), S33 (dislocation, sprain and strain of joints and ligaments of lumbar spine and pelvis) and S34 (injuries of nerves and lumbar spinal cord at abdomen, lower back and pelvis level).
- *Neck*; M40-M54 (dorsopathies), M80–M85 (disorders of the bone density and structure), M99 (biomechanical lesions, not elsewhere classified), S12 (fracture of neck), S13 (dislocation, sprain and strain of joints and ligaments at neck level) and S14 (injuries of nerves and spinal cord at neck level).
- *Shoulder;* M19 (other arthrosis), M60-M63 (disorders of muscles), M65-M68 (disorders of synovium and tendon), M70 (soft tissue disorders related to use, overuse and pressure), M71 (other bursopathies), M75 (shoulder lesions), M79 (other soft tissue disorders, not elsewhere classified), M99 (biomechanical lesions, not elsewhere classified), S42 (fracture of shoulder and upper arm), S43 (dislocation, sprain and strain of joints and ligaments of shoulder girdle) and S44 (injuries of nerves at shoulder and upper arm level).

## Appendix 2

**Table 3:** Associations (OR) between potential predictors and secondary care contacts in relation to diseases of the musculoskeletal system and connective tissue (*N* = 759)

Potential predictor	Contacts		Crude	Adjusted^**c**^	
	Cases^b^ (n)	%	OR	OR	95% CI
**Clinical**					
Pain 0-10^a^	112	15	1.23	1.24	(1.11;1.40)*
Standardized disability 0-100^a^	108	14	1.01	1.01	(1.00;1.02)*
Pain site					
Low back	57	17	1.00	1.00	–
Neck	23	11	0.60	0.69	(0.39;1.19)
Shoulder	32	14	0.80	0.79	(0.47;1.33)
**Socio-demographic**					
Level of education					
None	20	15	1.00	1.00	–
Low (< 3 years) or vocational and training	60	50	1.01	1.02	(0.59;2.00)
Middle (3–4 years) or high (> 4 years)	31	13	0.83	0.88	(0.45;1.71)
Sickness leave^d^					
No	95	14	1.00	1.00	–
Yes	16	20	1.44	1.60	(0.82;3.11)
Private Health Insurance					
No	74	15	1.00	1.00	–
Yes	30	13	0.85	0.94	(0.57;1.55)
**Psychological**					
Fear avoidance 0-20^a^	108	14	1.07	1.05	(1.01;1.10)*
Psychological wellbeing (WHO-5) 0-100^a^	112	15	0.98	0.98	(0.97;0.99)*
**General**					
General Health					
Excellent/Very good	40	13	1.00	1.00	–*
Good	45	14	1.11	1.16	(0.70;1.91)
Fair/poor	25	19	1.58	1.86	(1.00;3.45)

*Abbreviations*: *OR* Odds ratio, *CI* Confidence interval, *WHO* World Health Organization

* *P* < 0.05

^a^OR per 1 unit increase in scores

^b^patients with ≥1 contact to secondary care

^c^adjusted for age, sex, duration of pain and comorbidity

^d^Patients retired or with flex job or on disability pension was also included in this analysis (*n* = 186)

## Abbreviations

CI: Confidence Interval
ICD: International Classification of Diseases and Related Health Problems
IQR: Interquartile Range
NDI: Neck Disability Index
NHSR: National Health Service Register
NPR: National Patient Register
NPRS: Numeric Pain Rating Scale
OR: Odds Ratio
PASS: Patient Acceptable Symptom State
Quick DASH: Disabilities of the Arm, Shoulder and Hand
RMQ: Roland Morris Questionnaire
SD: Standard deviation
WHO: World Health Organization
ÖMPSQ: Örebro Musculoskeletal Pain Screening Questionnaire
